# Ambulatory ECG-based T-wave alternans and heart rate turbulence can predict cardiac mortality in patients with myocardial infarction with or without diabetes mellitus

**DOI:** 10.1186/1475-2840-11-104

**Published:** 2012-09-06

**Authors:** Ren Li-na, Fang Xin-hui, Ren Li-dong, Gong Jian, Wang Yong-quan, Qi Guo-xian

**Affiliations:** 1 Department of Cardiology, The First Affiliated Hospital of China Medical University, Shenyang, 110001, China; 2 Department of Anesthesiology, Central Hospital of Dalian, No. 826 Southwest Road, Shahekou District, Dalian, 116033, China; 3 Department of Clinical Pharmacy, School of Life Science and Biopharmaceutics, Shenyang Pharmaceutical University, 103 Wenhua Road, Shenyang, 110016, China

**Keywords:** Ambulatory electrocardiograms, Heart rate turbulence, Myocardial infarction, T-wave alternans, Diabetes mellitus

## Abstract

**Background:**

Many patients who survive a myocardial infarction (MI) remain at risk of sudden cardiac death despite revascularization and optimal medical treatment. We used the modified moving average (MMA) method to assess the utility of T-wave alternans (TWA) and heart rate turbulence (HRT) as risk markers in MI patients with or without diabetes mellitus (DM).

**Methods:**

We prospectively enrolled 248 consecutive patients: 96 with MI (post-MI patients); 77 MI with DM (post-MI + DM patients); 75 controls without cardiovascular disease (group control). Both TWA and HRT were measured on ambulatory electrocardiograms (AECGs). HRT was assessed by two parameters ─ turbulence onset (TO) and turbulence slope (TS). HRT was considered positive when both TO ≥0% and TS ≤2.5 ms/R-R interval were met. The endpoint was cardiac mortality.

**Results:**

TWA values differed significantly between MI and controls. Post-MI + DM patients had higher TWA values than post-MI patients (58 ± 21 μV VS 52 ± 18 μV, *P* = 0.029). Impaired HRT--increased TO and decreased TS were observed in MI patients with or without DM. During follow-up of 578 ± 146 days, cardiac death occurred in ten patients and three of them suffered sudden cardiac death (SCD). Multivariate analysis determined that a HRT-positive outcome [HR (95% CI): 5.01, 1.33–18.85; *P* = 0.017], as well as the combination of abnormal TWA (≥47 μV) and positive HRT had significant association with the endpoint [HR (95% CI): 9.08, 2.21–37.2; *P* = 0.002)].

**Conclusion:**

This study indicates that AECGs-based TWA and HRT can predict cardiac mortality in MI patients with or without DM. Combined analysis TWA and HRT may be a convenient and useful method of identifying patients at high risk for cardiovascular death.

## Background

Numerous studies over the past decades have provided a sound scientific basis for employing T-wave alternans (TWA), monitored during exercise testing, pacing, daily activities or from defibrillator electrogram as an index of vulnerability to ventricular arrhythmias and sudden cardiac death, as it represents an increased heterogeneity of ventricle repolarization on a beat-to-beat basis, and may provide a substrate for reentry [1-6]. Currently, there are both frequency domain- and time domain-based analysis methods of determining TWA. Our study used a time domain-based modified moving average (MMA) to analyze TWA measured on ambulatory electrocardiograms (AECGs) [7,8].

Following a premature ventricular contraction, there is a compensatory sinus pause, followed by sinus acceleration that typically overshoots the baseline heart rate before gradually decelerating back to baseline. This sequence of cycle length change has been labeled ‘heart rate turbulence’ (HRT). Abnormal findings for HRT are defined as TO ≥0% and TS ≤2.5 ms/RR interval [9]. Specifically, blunted post-premature ventricular contraction, sinus acceleration (less negative TO), and reduced subsequent deceleration (lower TS) have been introduced as an autonomic risk stratification marker for cardiac events after MI or ischemic cardiomyopathy [9-12].

As is well known, diabetes mellitus (DM) not only causes coronary artery disease and cardiac neuropathy, but also increases the risk of cardiac death in post-MI patients. Numerous studies have since established definitively that hyperglycemia is highly prevalent and that it is associated with an increased risk of death and in-hospital complications in patients with MI [13,14].

In our study, we prospectively assessed the utility of TWA and HRT, measured from 24-hour AECGs, in identifying the risk of cardiac mortality in post-MI patients with or without DM.

## Methods

### Patient population

Between April 2009 and December 2010 we prospectively enrolled 248 consecutive patients at the Department of Cardiology, the First Affiliated Hospital of China Medical University, 25 of them have been previously included in our prior study [15]. The population included 96 with MI (post-MI patients); 77 with MI with DM (post-MI + DM patients); and 75 controls (group control). The control group included patients with palpitations who underwent various noninvasive tests, such as 12-lead standard resting electrocardiogram (ECG), conventional echocardiographic and treadmill exercise tests, but were proven negative for organic heart disease. The patients had a 24-hour AECGs 1 to 3 weeks after hospitalization. Patients did not withhold the beta-blockers while testing TWA. Left ventricular ejection fraction (LVEF) was assessed by echocardiography. The diagnosis of MI was based on clinical course, serum creatine kinase levels, and ECG findings of ST-segment elevation. Diabetes mellitus was defined as a morning-fasting glucose of ≥126 mg/dL and hemoglobin A1c ≥6.5%, or use of antidiabetic medication. Written informed consent was obtained from each patient before enrollment. All study subjects underwent a full clinical examination and history, and any concurrent diseases and medications were recorded. Patients with atrial flutter or fibrillation, a cardiac pacemaker, atrioventricular block or bundle-branch block, frequent extrasystoles, and severe hepatic or renal disease were excluded.

### T-wave alternans

Analyses of modified moving average-based TWA were performed using the MARS PC system (GE Healthcare Inc, Milwaukee, WI, USA) running software version 7.03. Investigators who analyzed TWA and HRT were blinded to clinical characteristics and outcomes. TWA was analyzed from three channel records (V1; NASA,V3; CM3,V5;CM5) using AECGs measured as the peak difference in TWA amplitude between odd- and even-numbered beats at maximum heart rates (<120 beats/min). The MARS PC software identified periods of possible TWA using the MMA algorithm, a time domain-based method that bifurcates the beat stream and generates separate moving-average templates for odd versus even beats [7]. Average values were updated by a weighting factor of one-eighth difference between the ongoing average and the current pair of beats. TWA magnitude was analyzed as a continuous variable and determined for each 15 s of data. An additional algorithm minimized the effects of noise and artifacts, and noise limits of 20 μV were adopted in the system configuration. We overread and verified reported TWA values using the templates, which not only allow verification of TWA level but also allow investigators to discard TWA values contaminated by noise or artifact. Manual editing was performed if the data were ineligible due to noise or artifacts. The max TWA value was defined as the highest TWA value in any channel. In our study, TWA ≥ 47 μV was considered positive, based on previous reports [16].

### Heart rate turbulence

HRT was measured automatically using an algorithm applied to 24-hour AECGs obtained using the MARS Holter system (GE Healthcare Inc., Milwaukee, WI, USA). HRT parameters included turbulence onset (TO) and turbulence slope (TS), which were determined according to a previously published method [9]. The two phases of HRT are quantified by two numeric descriptors, TO and TS. TO is calculated as: TO = 100* ((RR1 + RR2) -(RR-2 + RR-1)) /(RR-2 + RR-1) where RR-2 and RR-1 are the two RR intervals immediately preceding the VPC coupling interval, and RR1 and RR2 are two RR intervals immediately after the compensatory pause. TS is defined as the maximum positive regression slope assessed over any 5 consecutive sinus rhythm RR intervals within the first 15 sinus rhythm RR intervals after the VPC. HRT values are usually classified into three categories: HRT Category 0: both TO and TS are normal (TO <0% and TS >2.5 ms/RR interval); HRT Category 1: either TO or TS is abnormal; HRT Category 2: both TO and TS are abnormal [17].

### Follow-up and study endpoints

All patients were followed as outpatients at our institute. Regular follow-up contact was obtained at a hospital visit every 2 or 3 months. When patients did not visit for more than 3 months, our medical staff conducted telephone interviews. The endpoint was prospectively defined as cardiac mortality. In patients who died, the causes were verified from the hospital, and from either the primary physicians or those who had witnessed the death. Patients who died of noncardiac causes such as stroke and cancer were not included in the endpoint and were excluded from analysis.

### Statistical analysis

All analyses were carried out using SPSS software, version 12.0 (SPSS Inc., Chicago, IL, USA). Data are presented as the mean ± SD or n (%). The statistical significance of differences between groups was determined by chi-square tests for discrete variables and t-tests for continuous variables. For analysis of the association between the endpoints and the clinical factors, Multivariate models to predict events for all patients were developed by using Cox proportional hazards regression. This Cox model included the following variables: age, sex, TWA, category of HRT, the presence of DM, LVEF and interaction of TWA and HRT. All reported P values are 2-tailed with an alpha level of 0.05 indicating statistical significance. A *P* value <0.05 was considered statistically significant.

## Results

### Patient characteristics

There were no significant differences among the three groups in age, hypertension and laboratory data. The LVEF differed significantly between controls and post-MI with or without DM patients (*P* < 0.05). There were no differences between post-MI and post-MI + DM patients in the sites of infarction or the coronary revascularization procedures (Table 1).

**Table 1:** Characteristics of enrolled patients twa values and HRT parameters

	**Post-MI**	**Post-MI + DM**	**Group control**	
	**(N = 96)**	**(N = 77)**	**(N = 75)**	***P*value**
Male gender	61(63.5%)	48(62.3%)	46(61.3%)	P = 0.96
Age(years)	65 ± 10	66 ± 8	62 ± 9	P = 0.65
Hypertension	42(43.6%)	36(46.8%)	31(41.3%)	P = 0.79
Heart Rate(bpm)	70 ± 9	69 ± 8	71 ± 9	P = 0.72
Medical therapies				
ACEI/ARB	75(78.1%)*	62(80.5%)*	10(13.3%)	P < 0.05
β-blockers	74(77.1%) *	60(77.9%)*	8(9.4%)	P < 0.05
Statins	81(84.4%) *	64(83.1%)*	11(14.6%)	P < 0.05
Site of infarction				
Anterior	33(34.4%)	25(32.5%)	–	P = 0.29
Lateral	29(30.2%)	27(35.1%)	–	P = 0.79
Inferior	34(35.4%)	25(32.5%)	–	P = 0.24
Coronary intervention	72(75.0%)	56(72.7%))	_	P = 0.74
Coronary bypass surgery	14(14.6%)	12(15.6%))	_	P = 0.69
LVEF (%)	46 ± 9*	45 ± 8*	59 ± 5	P < 0.05
TWA(μV)	52 ±18*	58 ± 21*†	37 ± 13	P < 0.05
TO(%)	-0.71 ± 2.17*	-1.03 ± 3.12*	-2.43 ± 1.71	P < 0.05
TS(ms/RRI)	4.96 ± 4.75*	4.60 ± 4.71*	13.03 ± 4.84	P < 0.05

* Post-MI patients with or without diabetes vs group control,*P* < 0.05.† Post-MI patients vs post-MI + DM patients, *P* < 0.05. ACEI/ARB, angiotensin-converting enzyme inhibitor/angiotensin-receptor blocker; LVEF, left ventricular injection fraction; TWA, T-wave alternans; HRT, heart rate turbulence**;** TO, turbulence onset; TS, turbulence slope.

### T-wave alternans

**Figure 1:**
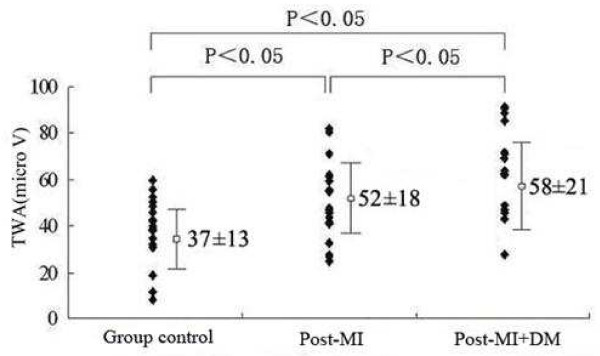
**T-wave alternans values in the three groups: Highest in post-MI + DM patients and lowest in group control.** Significant differences were observed within the groups by multiple comparisons.

**Figure 2:**
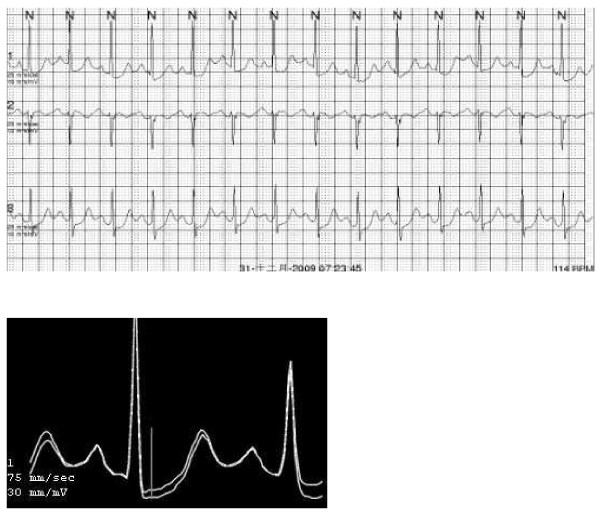
Superimposed MMA waveforms for the maximum TWA in V1 (70 μV) and the associated AECGs strip for a patient. The patient suffered SCD after 12 months.

The highest TWA values were found, as expected, in the post-MI + DM patients, while the lowest were found in the group control. There were significant differences in TWA values among the three groups (*P* < 0.05). The TWA values in post-MI + DM patients was higher than that in post-MI patients (*P* = 0.029) (Figure 1). A figure illustrating TWA in the AECGs of one of the patients (Figure 2).

### Heart rate turbulence

The average values for TO and TS differed significantly between group control and the other two groups (Table 1). HRT Category 0 had the highest incidence in the group control. HRT Category 2 was higher in the post-MI patients (abnormal in 20 patients [20.8%]) than in group control, while the highest incidence was in the post-MI + DM patients (abnormal in 18 patients [23.4%]) (Table 2).

**Table 2:** Distribution of HRT category in the three groups

	**Post-MI**	**Post-MI + DM**	**Group control**	***P*value**
	**(N = 96)**	**(N = 77)**	**(N = 75)**	
Category 0	42*	32 *	63	*P* < 0.05.
Category 1	34*	27*	11	*P* < 0.05.
Category 2	20*	18*	1	*P* < 0.05.

* Post-MI or post-MI + DM patients vs group control, *P* < 0.05

HRT, heart rate turbulence.

### Positive results of TWA and HRT

**Figure 3:**
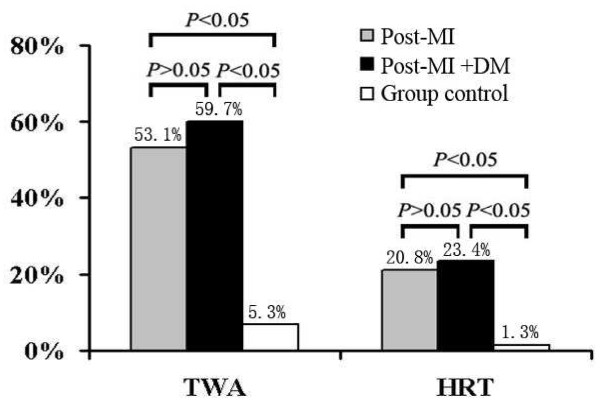
Positive Results of TWA (≥47 μV) and HRT (TO ≥0% and TS ≤2.5 ms/RRI):Post-MI with or without DM patients had a higher association of positive results for both TWA and HRT.

**Figure 4:**
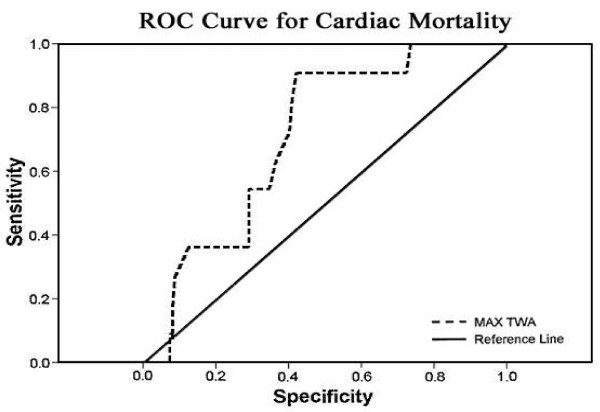
ROC curve for T WA magnitude and Cardiac mortality.

Post-MI patients with or without diabetes had a higher association of positive results for both TWA (≥47 μV) and HRT (TO ≥0% and TS ≤2.5 ms/RRI) compared with the group control (*P* < 0.05) (Figure 3). ROC curves for maximum TWA and cardiac death. Area under the curve was 0.708 (*P* = 0.021) (Figure 4).

### Endpoint results during the follow-up period

During a mean follow-up period of 578 ± 146 days, ten patients reached the cardiac mortality endpoint (four in post-MI patients, six in post-MI + DM patients). It was witnessed that three patients had SCD (two in post-MI patients, one in post-MI + DM patients). There was no difference between MI patients with or without DM who reached the endpoint. Two patients suffered noncardiac death (i.e., stroke, liver cancer) and were excluded from analysis. Multivariate analysis determined that a HRT-positive outcome [HR (95% CI): 5.01, 1.33- 18.85; *P* = 0.017], as well as the combination of abnormal TWA and positive HRT had significant association with the endpoint [HR (95% CI): 9.08, 2.21-37.2; *P* = 0.002)] (Table 3).

**Table 3:** Hazard ratios for the capacity of the variables to predict the development of the endpoint (cardiac mortality)

**Variables**	**Hazard ratio (95% CI)**	***P*value**
DM (yes/no)	0.36 (0.09—1.39)	0.14
LVEF(<40%)	1.04 (0.95—1.13)	0.44
HRT (positive)	5.01 (1.33—18.85)	0.02
TWA (≥47 μV)	7.44 (0.82—67.59 )	0.08
TWA + HRT (TWA ≥ 47 μV + HRT positive)	9.08 (2.22—37.8)	0.002

CI: confidence intervals; DM, diabetes mellitus; LVEF, left ventricular injection fraction; HRT, heart rate turbulence; TWA, T-wave alternans; TWA + HRT, TWA combined with HRT.

## Discussion

Patients with a positive microvolt-level TWA and HRT are characterized by an increased risk of ventricular tachyarrhythmias and cardiac mortality. We observed that TWA was elevated in patients following MI as our prior study [15]. Our data showed that patients in post MI with DM have higher TWA values than those without DM. MI patients with or without DM had higher association with positive results for TWA and HRT. A HRT-positive outcome, as well as the combination of abnormal TWA and positive HRT had significant association with cardiac mortality during the follow-up period.

Our study found that TWA was elevated in patients following MI, HRT parameters-TO and TS were significantly impaired in MI patients. A HRT-positive outcome, as well as the combination of abnormal TWA and positive HRT had significant association with the endpoint. Recently, the Consensus Guideline about TWA by International Society for Holter and Noninvasive Electrocardiology concluded the utility of TWA [18]. To date, over 100 studies enrolling a total of more than 12,000 patients support the predictive of TWA testing (including the frequency-domain Spectral Method and the time-domain Modified Moving Average method) for cardiovascular mortality and SCD. The HRT evaluation has thus been found appropriate in risk stratification after acute myocardial infarction (AMI), risk prediction, and monitoring of disease progression in heart failure, as well as in several other pathologies [12]. It is worth mentioning that in the REFINE (Risk Estimation Following Infarction Noninvasive Evaluation) trial they investigated the capacity of combined assessment of autonomic tone and cardiac electrical substrate to predict the development of serious outcomes after MI, and found that patients with impaired HRT plus abnormal Holter TWA at 10 to 14 weeks after MI were at higher risk for cardiac death or cardiac arrest, death from any cause, and fatal or nonfatal cardiac arrest [19].

Diabetes mellitus (DM) is a well-established risk factor for ischemic heart disease. Insulin resistance is a precursor and a characteristic feature of T2DM and it is also associated with higher risk of cardiovascular diseases [20,21]. Rodríguez-Colón et al. reported that among persons with T2DM have more severe form of insulin resistance, and the circadian mechanisms of cardiac autonomic modulation are impaired [22]. Prognosis after MI is worse in patients with DM as compared to patients without DM [13]. Autonomic nervous dysfunction is the main complication of DM and associated with an increased risk of mortality in patients with diabetes and survivors of myocardial infarction [23]. In our prior study, TWA was moderate correlated with heart rate variability (HRV), and seemed to be associated with the sympathetic component of the autonomic nervous system [15]. Elevated R wave amplitudes, widening of QTc intervals and decreased HRV in an electrocardiogram (ECG) are early markers of diabetic autonomic neuropathy. The severity of diabetic autonomic neuropathy has a direct relationship with mortality risk. VanHoose et al. found that aerobic exercise training may attenuate the ECG changes, included R wave amplitudes, QTc intervals and HRV in the model of T2DM-Zucker Diabetic Fatty rat [24]. Nieminen et al. found a significant increase in TWA among diabetics in the FINCAVAS (The Finnish Cardiovascular Study) population of 1,037 consecutive patients [16]. The prevalence of DM was the only parameter that differed among the groups with statistical significance. Molon et al. have shown that in patients with DM and no known cardiovascular disease, abnormal TWA prevalence was a very common condition (approximately 25%) among people with T2DM without manifest cardiovascular disease and was closely correlated to glycemic control [25,26]. In multivariate regression logistic analysis, HbA1c (OR = 13.6, 95% CI =2.0-89.1) predicted abnormal TWA independent of other potential confounders. Jouven et al. conducted a population-based case–control study at the Group Health Cooperative [27]. Cases (n = 2040) experienced out-of-hospital cardiac arrest due to heart disease. These investigators observed that a progressively higher risk of SCD was associated with borderline diabetes (OR = 1.24, 95% CI = 0.98-1.57), diabetes without microvascular disease (OR = 1.73, 95% CI = 1.28-2.34), and diabetes with microvascular disease (OR = 2.66, 95% CI = 1.84-3.85). Bonapace et al. studied 50 consecutive, well controlled T2DM outpatients without a history of ischemic heart disease and with normal systolic function [28]. All patients underwent a complete echocardiographic Doppler evaluation with spectral tissue Doppler analysis. TWA analysis was performed noninvasively during submaximal exercise. Multivariable logistic regression analysis revealed that higher E/e′ ratio was the only independent correlate of abnormal TWA (OR = 3.52, 95% CI = 1.19-10.6) after controlling for glycemic control and other potential confounders. They found that early diastolic dysfunction is independently associated with TWA abnormality in T2DM individuals with normal systolic function. Hennersdorf et al. found that abnormal TWA was found in 33% of patients with both hypertension and left ventricular hypertrophy (LVH) [29]. Hakalahti et al. investigated the possible association between the beta-1 adrenergic receptor (b1AR) Arg389Gly polymorphism (which plays a fundamental role in the regulation of cardiovascular functions) and LVH among non-diabetic and diabetic AMI survivors [30]. They found that the b1AR Arg389 variant seem to confer higher risk of developing LVH among non-diabetic patients who have suffered AMI. But it does not exist among diabetic AMI survivors. They hypothesized that this negative finding is caused by the strong association between DM and LVH, which may mask the presumably weaker effect of the b1AR Arg389 variant on the left ventricular structure.

Miwa et al. assessed the utility of HRT as a risk marker in post-MI patients with and without DM [31]. They prospectively enrolled 231 consecutive DM patients and 300 non-DM patients after acute MI. HRT was considered positive when both TO ≥0% and TS ≤2.5 ms/RRI criteria were met. The endpoint was defined as cardiac mortality. Forty-two of 222 patients (19%) were HRT positive. During follow-up of 876 ± 424 days, 26 patients (22%) reached the endpoint. HRT- positive outcome had significant value with a hazard ratio of 3.5 (95% CI = 1.4-8.8). Stein et al. investigated 481 hospitalized patients (24% of whom had DM) after AMI with HF and left ventricular dysfunction [32]. Over a 1-year follow-up, 55 died, 49 of cardiovascular causes. The study showed that abnormal HRT predicted cardiovascular mortality in high-risk patients with AMI and left ventricular dysfunction. Bauer et al. found a higher percentage of abnormal values for both HRT parameters in patients with DM than without DM in a subgroup analysis [33]. Barthel et al. enrolled 1455 survivors of an a AMI who were in sinus rhythm [17]. The primary endpoint was all-cause mortality. During a follow-up of 22 months, 70 patients died. These investigators showed that the combined assessment of HRT, LVEF ≤ 30%, age ≥ 65 years, and DM can predict the population at high risk. Thus, abnormal HRT is associated with DM in post-MI patients, and HRT can detect cardiac neuropathy due to DM. Balcioğlu et al. investigated the detection of diabetic neuropathy in patients with T2DM and no obvious heart disease by autonomic markers that included HRT and HRV, and found that HRT was the most powerful indicator of cardiac diabetic neuropathy [34]. Thus, HRT might be more sensitive indicators of cardiac autonomic abnormality in post-MI patients with DM because these patients have disturbance of both parasympathetic and sympathetic tone.

However, negative studies have also appeared. Martin et al. performed a case–control cross-sectional study in 140 patients [35]. Patients performed a symptom-limited Bruce protocol exercise test with assessment of TWA by the spectral method. Logistic regression analysis in all patients showed that abnormal TWA was not related to prevalent DM (OR = 0.9, 95% CI =0.4–1.8). Jackson et al. investigated the utility of TWA treadmill testing in an unselected population of patients with HF and found that half of patients with HF are eligible for TWA testing and the most common result is an indeterminate test [36]. It concluded that TWA treadmill testing is not widely applicable in typical HF patients and is unlikely to refine risk stratification for sudden death. Wijers et al. summarized the main reason for indeterminate test of TWA treadmill testing [37]. They reported that inability to achieve the target heart rate is the main reason for an indeterminate result of TWA testing. Therefore, it is questionable whether TWA alone is able to be of predictive value and for what patient population. But they concluded that combine a number of invasive and non-invasive electrophysiological risk markers (including TWA and short-term variability) could be the solution.

### Study limitations

Because of the limited scope of our study, we did not include ventricular late potentials obtained from signal-averaged electrocardiogram, baroreflex sensitivity, and other electrocardiographic markers, all of which have been shown to be significant predictors in other populations. It is not known the value of these markers in predicting cardiac death or arrhythmic events in this study population.

## Conclusions

The results of this study indicated that patients with MI, especially those complicated with diabetes, have higher TWA values and abnormal HRT, and have a high risk of life threatening ventricular arrhythmias and cardiac death, possibly because of increased repolarization heterogeneity and abnormal autonomic function. Combined analysis using MMA-based TWA and HRT may be a convenient and useful method of predicting patients at high risk for arrhythmic events and cardiovascular death.

## Abbreviations

AECGs, Ambulatory electrocardiograms; b1AR, Beta-1 adrenergic receptor; DM, Diabetes mellitus; ECG, Electrocardiogram; HF, Heart failure; HRT, Heart rate turbulence; HRV, Heart rate variability; LVH, Left ventricular hypertrophy; MI, Myocardial infarction; MMA, Modified moving average; SCD, Sudden cardiac death; TO, Turbulence onset; TS, Turbulence slope; TWA, T-wave alternans.

## Competing interests

The authors declare no competing interests.

## Author contributions

RL, design and data collection and drafted the manuscript. FX and WY, data analysis. GJ and RL, statistics. QG, design, critical revision of article and approval of article. All authors read and approved the final manuscript.
